# Striving to provide universal health coverage in Kazakhstan

**DOI:** 10.2471/BLT.19.020419

**Published:** 2019-04-01

## Abstract

Four decades after the declaration of Alma Ata, Kazakhstan still struggles to provide basic health care to its citizens. This may now be changing. Andrey Shukshin reports.

Lyubov Petrova (name changed at her request), a 73-year-old pensioner who lives in the city of Kapchagay, Kazakhstan, gets annoyed when people talk to her about the Declaration of Alma Ata. And the topic tends to come up, since the declaration calling for universal health access was adopted in Alma Ata (now Almaty), a city just 80 km south of where she lives.

“Don’t talk to me about universal health coverage,” she says. “In Kazakhstan you get it only if you are ready to pay for it yourself.”

Lyubov has breast cancer and is not alone in having to dig deep into her own pockets for health care she cannot afford. According to Zarina Temekova, head of the Centre for Healthcare Economics at the Republican Centre for Health Development (RCHD) in the Kazakh capital, Astana, out-of-pocket payments account for about 38% of all health-care spending in the country.

High out-of-pocket expenditure tends to be associated with low government health expenditure, and in Kazakhstan, public expenditure on health is very low. At just 2% of gross domestic product (GDP), Kazakhstan has less than a third of the 6.5% average public expenditure on health reported by Organisation for Economic Co-operation and Development (OECD) countries.

This may be about to change. “There is high expectation that the government of Kazakhstan will substantially increase its spending on health in the near future,” says Bayarsaikhan Dorjsuren, Senior Health Systems Advisor at the World Health Organization.

That expectation derives in large part from recent statements made by the President of Kazakhstan, Nursultan Nazarbayev, including an address to the nation in October 2018, in which he called for the government to raise the total expenditure on health, education and science.

To support implementation of that proposed increase, the Ministry of Health drew up a draft budget for 2019–2023, taking into account expenditures on science and education. The draft budget indicated the need for additional funds to cover the cost of guaranteed free medical care, salary growth for medical workers, renewal of fixed capital, and medical services for which there is a growing need, including oncology, high-tech medical services, rehabilitation and palliative care.

How much money will eventually be channelled into funding these requirements is not entirely clear. However, according to Temekova, there is a preliminary agreement with the Ministry of National Economy for overall health-care expenditure (including 1.3% private expenditure) to rise from the current 3.3% to 4.7% of GDP before 2023, with all of the increase coming from additional government spending.

“There is a high expectation that the government of Kazakhstan will substantially increase its spending on health in the near future.”

Bayarsaikhan Dorjsuren

According to Nurbol Kuzembayev, Deputy Director at the RCHD, the extra public funding will come from general taxation. Funding will also come from a new national health insurance fund, which started collecting insurance premiums from employers in 2018 at a rate of 1% of monthly income in the initial year (incrementally rising thereafter).

The health insurance fund was due to start reimbursing medical services provided under the health insurance scheme in January 2018, but payments are now scheduled to begin in January 2020. Citizens and permanent residents of Kazakhstan will then have access to two packages of medical care, the state-guaranteed basic package and the health insurance fund package, including rehabilitation and palliative care. Unemployed and retired people will also be covered, the scheme thus aligning with Sustainable Development Goal 3.8, which sets a target of universal health coverage for all.

What exactly is to be included in these packages, however, has yet to be defined, reflecting the protracted debate around the issue.

President Nazarbayev signed off on the new state-guaranteed basic package in January. “The new package will include prevention, diagnosis and treatment for 30 of the most significant diseases, including: mental health conditions, substance abuse disorders, tuberculosis, cancer, HIV, diabetes and arterial hypertension,” says Kuzembayev, adding that services covered include ground and air ambulance services; outpatient consultations, diagnosis and treatment; and inpatient and palliative care. More details will emerge once legislation is enacted.

One of the two main aims of these reforms is to lower out-of-pocket payments, so that people like Lyubov can access the care they need without risking impoverishment.

On this front, prospects seem promising, given the resource mobilization initiatives that are under way. “It is assumed that the share of private spending in 2025 will be about 22-25%,” says Kuzembayev.

The second aim of the reforms is to ensure access to needed health services. Here the challenges are considerable, since, despite recent efforts to bolster the primary health care system, service delivery remains dominated by hospitals, which leaves people such as Lyubov struggling to access services.

“In Kapchagay you can get simple things like blood or urine tests through the public health system. Anything more complicated is either unavailable or requires a weeks-long wait,” she says.

Here too are signs of change. The current four-year State Health Development Programme includes a focus on boosting primary health care delivery, through private investment in health facility construction and upgrades.

To implement this programme, the Ministry of Health, working in collaboration with the local authorities, has developed regional plans, which define the infrastructure upgrade required, and called for 1.3 trillion tenge (US$ 3.4 billion), including private investment of approximately 800 billion tenge (US$ 2.1 billion), to be invested by public–private partnerships.

The government also plans to improve working conditions for doctors, decreasing the patient load from 2200 per general practitioner to 1700 by the end of 2019, and raising salaries to bring them to 250% of the national average wage (currently about US$ 5000) by 2023.

As important as increased investment of resources, is the change in health policy direction. “There is a commitment to move away from hospital-centric, curative care, to people-centred preventive care, delivered through a reinvigorated primary health care system,” says Kuzembayev, drawing attention to a disease management policy, which has been implemented since 2013, with programmes focused on diabetes and cardiovascular diseases.

Assessments of selected primary health care providers in 2013–2016 reported improved health outcomes, including stabilization of blood pressure among 75% of hypertension patients, lowering of glycosylated haemoglobin levels among 65% of diabetes patients, and a two-fold decrease in hospital admissions of patients with heart failure. To support these programmes, President Nazarbayev, announced that the salaries of general practitioners implementing the disease management programmes will be increased by 20%, in addition to the increase mentioned above.

“There is a commitment to move away from hospital-centric, curative care to people-centred preventive care delivered through a reinvigorated primary health care system.”

Nurbol Kuzembayev

Another important area of reform relates to the digitalization of health care. The Kazakhstan health system is going paperless by investing in informatics infrastructure. In January, Kazakhstan joined the group of countries using SNOMED (Systematized Nomenclature of Medicine), a computer-processable collection of medical terms that facilitates indexation, storage, retrieval, and aggregation of medical data across specialties and sites of care.

Finally, Kazakhstan is making a major push to bolster the technical capacity required to support the transition to universal health coverage. “The country has made great strides in terms of technical capacity in recent years, and is ambitious to achieve more,” says WHO’s Dorjsuren, drawing attention to the fact that Kazakhstan joined the WHO-hosted P4H Network, a collaborative health financing and economics platform for countries committed to developing UHC, in September 2017.

“Kazakhstan joined partly to open a dialogue with P4H experts, and partly to begin conversations with other members, including neighbouring countries,” Dorjsuren explains.

Temekova’s excitement regarding this development is palpable. “We are already holding talks with neighbour countries inviting them to join,” she says. “Being the only post-Soviet state on the P4H board, Kazakhstan is becoming a hub for promoting UHC in Russian-speaking countries.”

Such ambitions are laudable, but the road to Alma Ata is a challenging one and building it in Kazakhstan is going to require continued commitment from the highest levels of government, and – if no-one is to be left behind – an acute awareness of the fact that the needs of people like Lyubov Petrova are still not being met.

**Figure:**
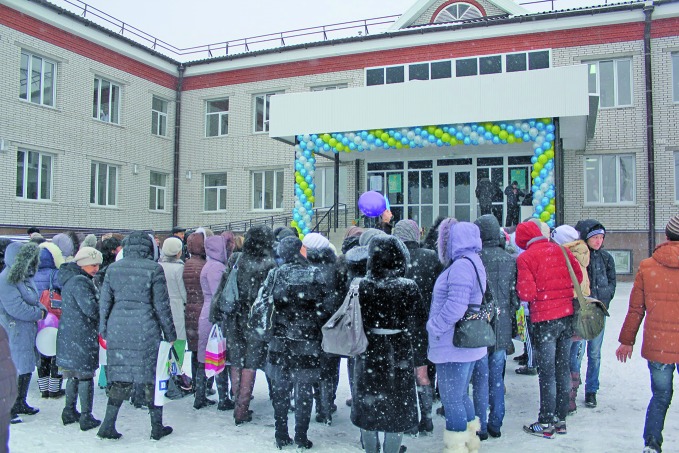
Crowd awaits opening of a city polyclinic in Uralsk, Kazakhstan

Courtesy of the Republican Centre for Health Development/Kazakhstan.

